# Long non-coding RNA *FEZF1-AS1* facilitates cell proliferation and migration in colorectal carcinoma

**DOI:** 10.18632/oncotarget.7168

**Published:** 2016-02-03

**Authors:** Na Chen, Dan Guo, Qiong Xu, Minhui Yang, Dan Wang, Man Peng, Yanqing Ding, Shuang Wang, Jun Zhou

**Affiliations:** ^1^ Department of Pathology, Nanfang Hospital, Southern Medical University, Guangzhou 510515, China; ^2^ Department of Pathology, School of Basic Medical Sciences, Southern Medical University, Guangzhou 510515, China; ^3^ Department of Pharmacy, Nanfang Hospital, Southern Medical University, Guangzhou 510515, China

**Keywords:** FEZF1-AS1, FEZF1, colorectal cancer, long non-coding RNA

## Abstract

Long non-coding RNAs (lncRNA) have been shown to play important roles in the development and progression of cancer. Here, we discovered a novel long noncoding RNA (lncRNA) *FEZF1* antisense RNA1 (*FEZF1-AS1*) is markedly upregulated in human primary colorectal carcinoma (CRC) and associated with CRC metastasis and poor prognosis. Moreover, the downregulation of *FEZF1-AS1* expression significantly inhibited the CRC cells proliferation, migration and invasiveness, suppressed S-phase entry *in vitro*, and repressed tumor growth and metastasis *in vivo*. In contrast, overexpression of *FEZF1-AS1* could promote the aggressive behaviors of CRC cells. We further discovered that the downregulation of *FEZF1-AS1* reduced its sense-cognate gene *FEZF1* mRNA and protein expression in CRC cells. There was a positive correlation between *FEZF1-AS1* and *FEZF1* expression in CRC. Moreover, *FEZF1* knockdown also significantly suppressed CRC cell proliferation, migration, and invasion. Our findings indicate that the dysregulation of *FEZF1-AS1* participates in colorectal tumorigenesis and progression, which might be achieved, at least in part, through *FEZF1* induction.

## INTRODUCTION

Colorectal carcinoma (CRC) is one of the most common cancers in the world. In China, CRC ranks fifth among cancer deaths and its incidence is increasing continuously [1]. Though alterations in oncogenes and tumour suppressor genes have been reported in CRC [2], the precise molecular mechanisms underlying CRC pathogenesis remain to be fully elucidated. The long noncoding RNAs (lncRNAs) are a class of transcribed RNA molecules over 200 nucleotides with no protein-coding capacity [3]. Through regulating gene expression, lncRNAs have been shown to play crucial roles in multiple biological processes such as development, differentiation and carcinogenesis [4–7]. Dysregulated lncRNA expression has been reported in various types of cancers, such as bladder, prostate, lung, breast, gastric and colorectal cancers [8–15], indicating that lncRNAs may be involved in tumorigenesis or tumor progression.

FEZ family zinc finger 1 antisense RNA 1 (*FEZF1-AS1*) which located on the opposite strand of gene *FEZF1*, is a recently identified long non-coding RNA. However, its function in normal or cancer cells remain unknowns. In this study, we first identified that the up-regulation of lncRNA *FEZF1-AS1* was associated with aggressive phenotypes of CRC and the poor prognosis in patients with CRC. Further function experiments revealed that *FEZF1-AS1* increase cell proliferation, migration and invasiveness. Moreover, *FEZF1*-*AS1* knockdown reduced its sense-cognate gene *FEZF1* mRNA and protein expression in CRC cells, and there was a positive correlation between *FEZF1-AS1* and *FEZF1* expression in CRC. Furthermore, we validated that *FEZF1* knockdown also significantly suppressed CRC cell proliferation, migration, and invasion. Taken together, these results suggest that *FEZF1-AS1* participates as a non-coding oncogene in CRC carcinogenesis and metastasis.

## RESULTS

### LncRNA *FEZF1-AS1* is up-regulated in human CRC tissues and associated with metastasis

**Figure 1:**
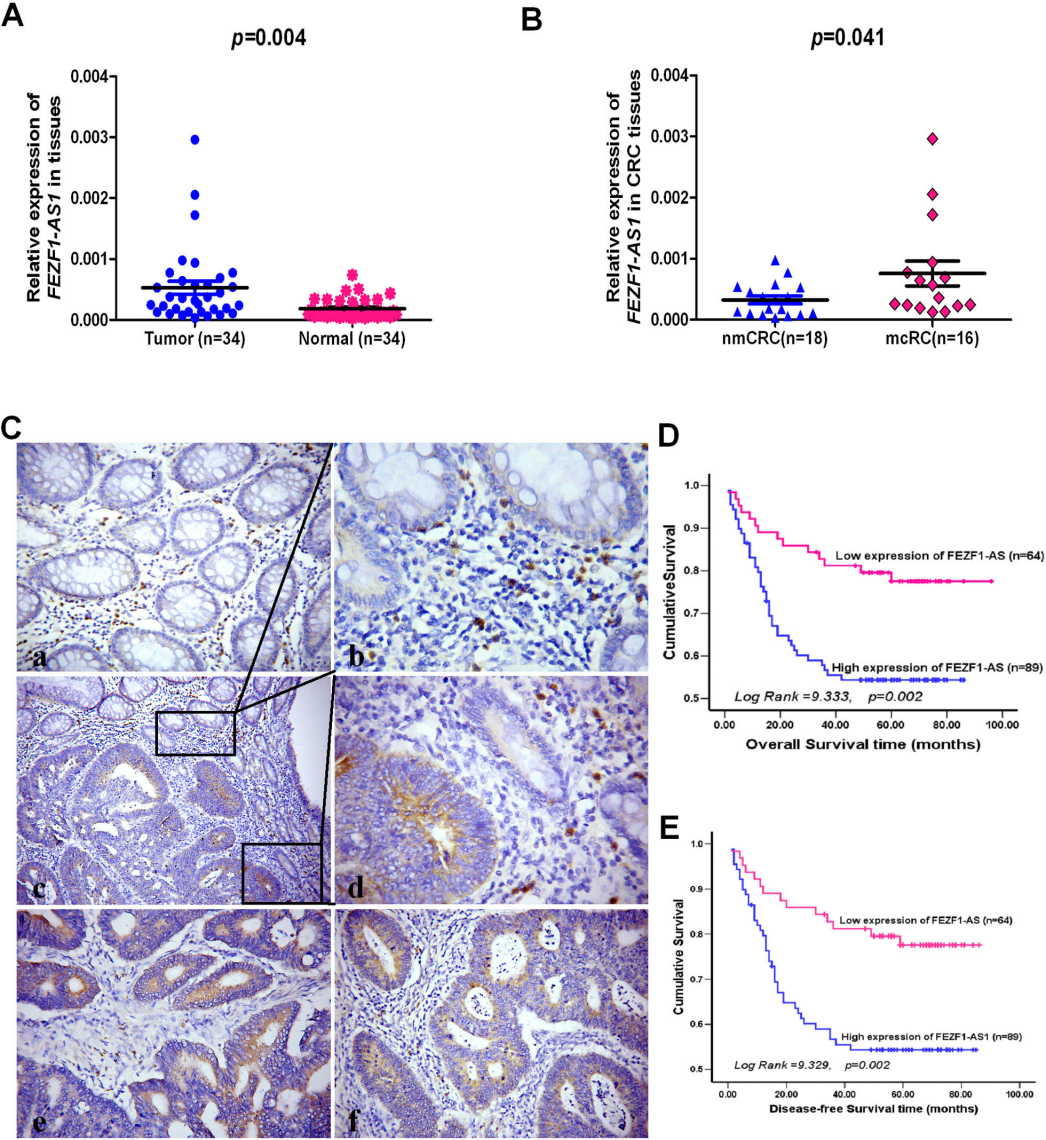
The levels of *FEZF1-AS1* expression in CRC tissues by qRT-PCR or *in situ* hybridization and its prognostic value in patients with CRC (**A**) Expression levels of *FEZF1-AS1* in paired CRC and adjacent normal tissues. (**B**) The *FEZF1-AS1* expression in CRC tissues with or without metastases. nmCRC denotes CRC tissues without metastases; mCRC denotes CRC tissues with lymph node metastases. (**C**) Expression analysis of *FEZF1-AS1* in normal colorectal mucosa and CRC tissues by *in situ* hybridization. (a) Negative expression of *FEZF1-AS1* in normal colorectal mucosa; (b–d) High expression of *FEZF1-AS1* in a tumor tissue sample and low or negative expression of *FEZF1-AS1* in its normal mucosal counterpart from the same patient were observed in one filed or two independent magnification fields. (e, f) High expression of *FEZF1-AS1* in CRC tissues; (**D, E**) Kaplan-Meier analysis of (D) overall and (E) disease-free survival in all patients with CRC according to *FEZF1-AS1* expression.

Using real-time RT-PCR, we detected the expression levels of *FEZF1-AS1* in 34 pairs of CRC and adjacent non-neoplastic mucosa tissues. The results indicated that *FEZF1-AS1* expression levels in tumor tissues of CRC patients were significantly higher than those in corresponding normal tissues (*p* = 0.004, Figure 1A). It was observed that the up-regulation of *FEZF1-AS1* in tumor samples was associated with lymph-node metastasis to a significant extent (*p* = 0.041, Figure 1B). In patients diagnosed with lymph node metastases, the relative mean expression of *FEZF1-AS1* was over 2.30 fold higher than that in patients without metastases (0.00076 ± 0.00020 *vs.*0.00033 ± 0.00006). This association indicated that *FEZF1-AS1* might have a pivotal role in CRC metastasis.

### Up-regulation of *FEZF1-AS1* is associated with aggressive phenotypes of patients with CRC

To explore whether *FEZF1-AS1* expression levels are associated with the clinicopathological factors of CRC, we measured *FEZF1-AS1* expression in a large cohort of 153 archived paraffin-embedded CRC and normal colon tissues using *in situ* hybridization. We found *FEZF1-AS1*-specific staining was observed in the cytoplasm of benign and malignant epithelial cells (Figure 1C). We also observed that 89/153 (58.17%) CRCs had high-level expression of *FEZF1-AS1*, whereas 22/56 (39.29%) normal mucosa tissues had high-level expression of *FEZF1-AS1* (*p* = 0.015). The correlation analysis between clinicopathological characteristics and *FEZF1-AS1* level indicated that high-level expression of *FEZF1-AS1* was significantly associated with T-stage (*p* = 0.005), lymph node metastasis (*p* = 0.004), and distant metastasis (*p* = 0.020) in patients with CRC. However, it was not associated with other clinical pathological features, including patients' age, sex, tumor site, tumor size, and tumor differentiation degree (*p* > 0.05, Table 1).

**Table 1:** Correlation between the clinicopathological features and expression of FEZF1-AS1

		FEZF1-AS1 expression		
Characteristics	*n*	low (%)	high (%)	*P* value
Gender				
Male	95	35 (36.8)	60 (63.2)	
Female	58	29 (50.0)	29 (50.0)	0.109
Age (years)				
< 50	73	32 (49.3)	37 (50.7)	
≥ 50	80	28 (35.0)	52 (65.0)	0.073
Tumor site				
Proximal colon	47	22 (46.8)	25 (53.2)	
Distal colon	37	16 (43.2)	21 (56.8)	
Rectum	69	26 (37.7)	437 (62.3)	0.607
Tumor size (cm in diameter)				
< 5	50	17 (34.0)	33 (66.0)	
≥ 5	103	47 (45.6)	56 (54.4)	0.171
Tumor differentiation				
Good	15	8 (53.3)	7 (46.7)	
Moderate	109	40 (36.7)	69 (63.3)	
Poor	29	16 (55.2)	13 (44.8)	0.128
T-stage				
1–2	22	16 (72.7)	6 (27.3)	
3	122	47 (38.5)	75 (61.5)	
4	9	2 (22.2)	7 (77.8)	0.005
N-stage				
0	92	47(51.1)	45 (48.9)	
1–2	61	17(27.9)	44 (72.1)	0.004
M-stage				
0	109	52(47.7)	57 (52.3)	
1	44	12(27.2)	32 (72.8)	0.020

### Up-regulation of *FEZF1-AS1* is correlated with poor prognosis of patients with CRC

To further evaluate the prognostic value of *FEZF1-AS1* in CRCs, we analyzed the association between *FEZF1-AS1* expression and survival duration using Kaplan-Meier analysis with the log-rank test. The results revealed that high-level expression of *FEZF1-AS1* in CRC was significantly correlated with overall survival (Log Rank = 9.333, *p =* 0.002, Figure 1D) and disease-free survival (Log Rank = 9.329, *p =* 0.002, Figure 1E) of CRC patients. The high-level of *FEZF1-AS1* was associated with short survival time (53.53 ± 3.86 *vs.*79.40 ± 4.02).

To determine whether the expression of *FEZF1-AS1* was an independent prognostic factor for CRC, univariate and multivariate analyses were performed. The results indicated that high-level expression of *FEZF1-AS1* was considered as an independent prognostic factor of outcomes in patients with CRC (*p* = 0.035, Table 2).

**Table 2:** Summary of overall survival analyses by univariate and multivariate COX regression analysis

	Univariate analysis			Multivariate analysis		
Variables	*P* value	HR	CI (95%)	*P* value	HR	CI (95%)
**Gender**	0.587	0.856	0.490–1.497			
**Age**	0.576	0.859	0.504–1.464			
**Tumor site**	0.834	0.967	0.707–1.322			
**Tumor size**	0.361	1.32	0.727–2.394			
**Tumor differentiation**	0.032	1.739	1.048–2.885			
**T-stage**	< 0.001	1.795	1.318–2.444	0.955	0.986	0.604–1.609
**N-stage**	< 0.001	2.887	1.674–4.979	0.66	0.825	0.351–1.943
**M-stage**	< 0.001	9.458	5.294–16.898	0.137	2.141	0.786–5.836
**FEZF1-AS1 expression**	0.003	2.495	1.356–4.590	0.035	2.401	1.065–5.411

### *FEZF1-AS1* promotes CRC cell proliferation and colony formation *in vitro*

**Figure 2:**
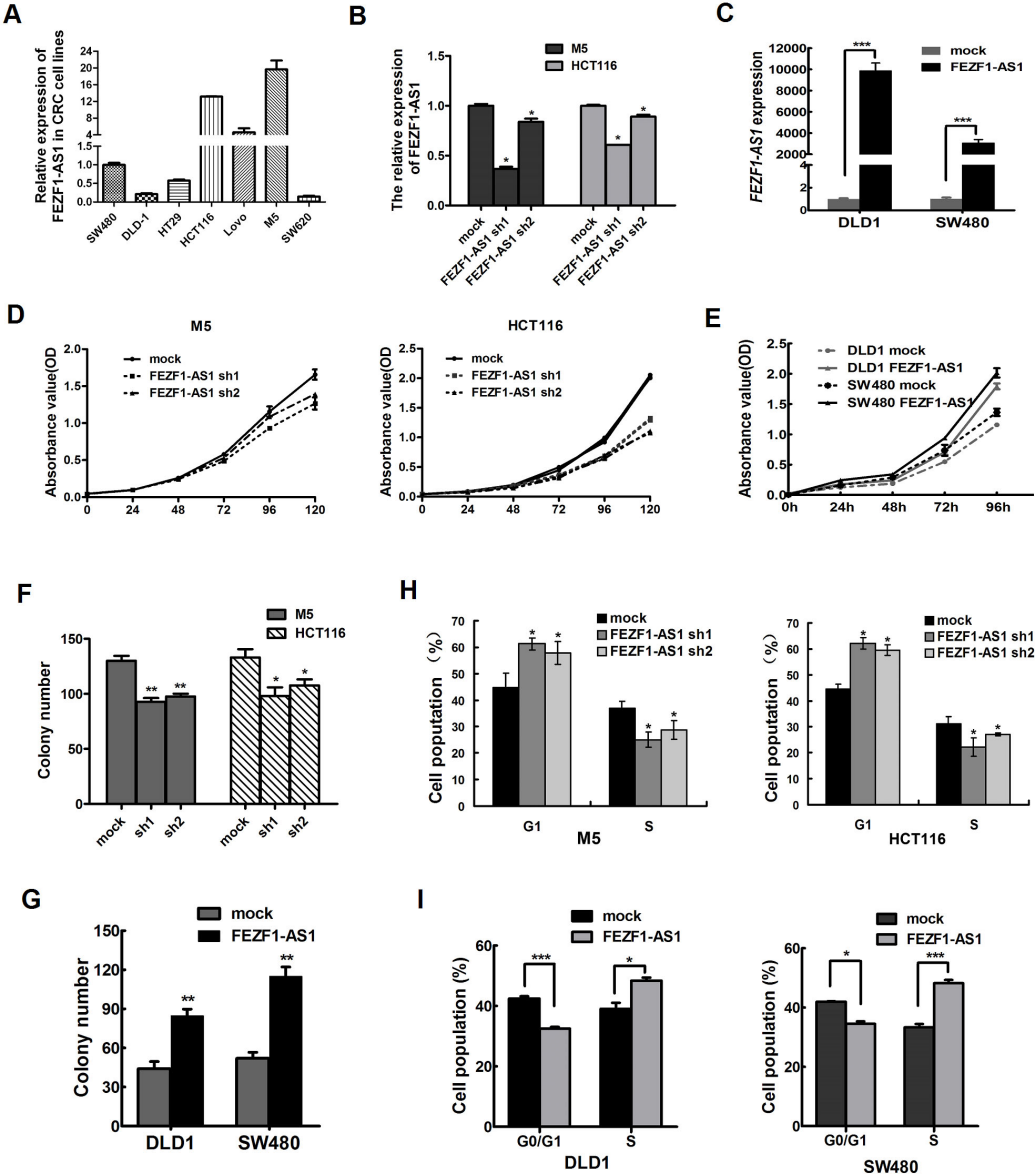
*FEZF1-AS1* promotes CRC cell proliferation *in vitro* (**A**) Expression of *FEZF1-AS1* in CRC cell lines and subclones by Real-time RT-PCR. (**B, C**) Detection of *FEZF1-AS1* expression level in CRC cells after shRNA-mediated knockdown of *FEZF1-AS1* or overexpression of *FEZF1-AS1* by qRT-PCR. (**D, E**) The effect of *FEZF1-AS1* on cell proliferation was evaluated by CCK-8 assay after knockdown or overexpression of *FEZF1-AS1* in CRC cells. (**F, G**) Colony-forming assays were performed to determine the growth of CRC cells. (**H**, **I**) Cell cycle was analyzed by flow cytometry. Data were presented as mean ± SD. The results were reproducible in three independent experiments. *p < 0.05, **p < 0.001,***p < 0.0001.

We measured the expression level of *FEZF1-AS1* in a panel of CRC cell lines (Figure 2A), and found that there were higher expression level of *FEZF1-AS1* in HCT116, M5 and LOVO cell line. So, we firstly inhibited the endogenous expression of *FEZF1-AS1* in M5 and HCT116 cells by shRNA to further investigate the biological effects of *FEZF1-AS1* on CRCs. Moreover, *FEZF1-AS1* was overexpressed in SW480 and DLD-1 cells using transfection of pcDNA-FEZF1-AS1 (*p* < 0.001, Figure 2B and 2C). The growth curves determined by CCK-8 assays indicated that cell proliferation was dramatically reduced by knockdown of endogenous *FEZF1-AS1* expression in M5 and HCT116 cells, whereas overexpression of *FEZF1-AS1* enhanced the proliferative capacity of SW480 and DLD-1 cells (*p* < 0.001, Figure 2D and 2E).

Further colony formation assays also showed that down-regulation *FEZF1-AS1* expression could significantly inhibit the colony formation M5 and HCT116 cells. In contrast, overexpression of *FEZF1-AS1* had the opposite effect (*p* < 0.05, Figure 2F and 2G). Thus, these data revealed that *FEZF1-AS1* enhances CRC cell growth.

### Knockdown of *FEZF1-AS1* promotes G1 arrest, but does not cause apoptosis in CRC cells

To probe potential mechanisms by which *FEZF1*-*AS1* enhanced CRC cell proliferation, we assessed cell cycle and apoptosis in CRC cell lines after *FEZF1*-*AS1* knockdown or overexpression. Flow cytometric cell cycle assays in M5 and HCT116 cells demonstrated that *FEZF1-AS1* knockdown led to a significant accumulation of cells at G0/G1-phase and a significant decrease in cells in S-phase (*p* < 0.05, Figure 2H). Conversely, the overexpressed *FEZF1*-*AS1* mainly led to a reduction in the G0/G1 population and an increase in the S-phase (*p* < 0.05; Figure 2I). However, the proportion of apoptotic cells remained similar (data not shown). Thus, *FEZF1-AS1*-induced promotion of CRC cells proliferation appeared to be mediated by modulation of the G1-S checkpoint, rather than by apoptosis.

### *FEZF1-AS1* promotes CRC cells migration and invasion *in vitro*

**Figure 3:**
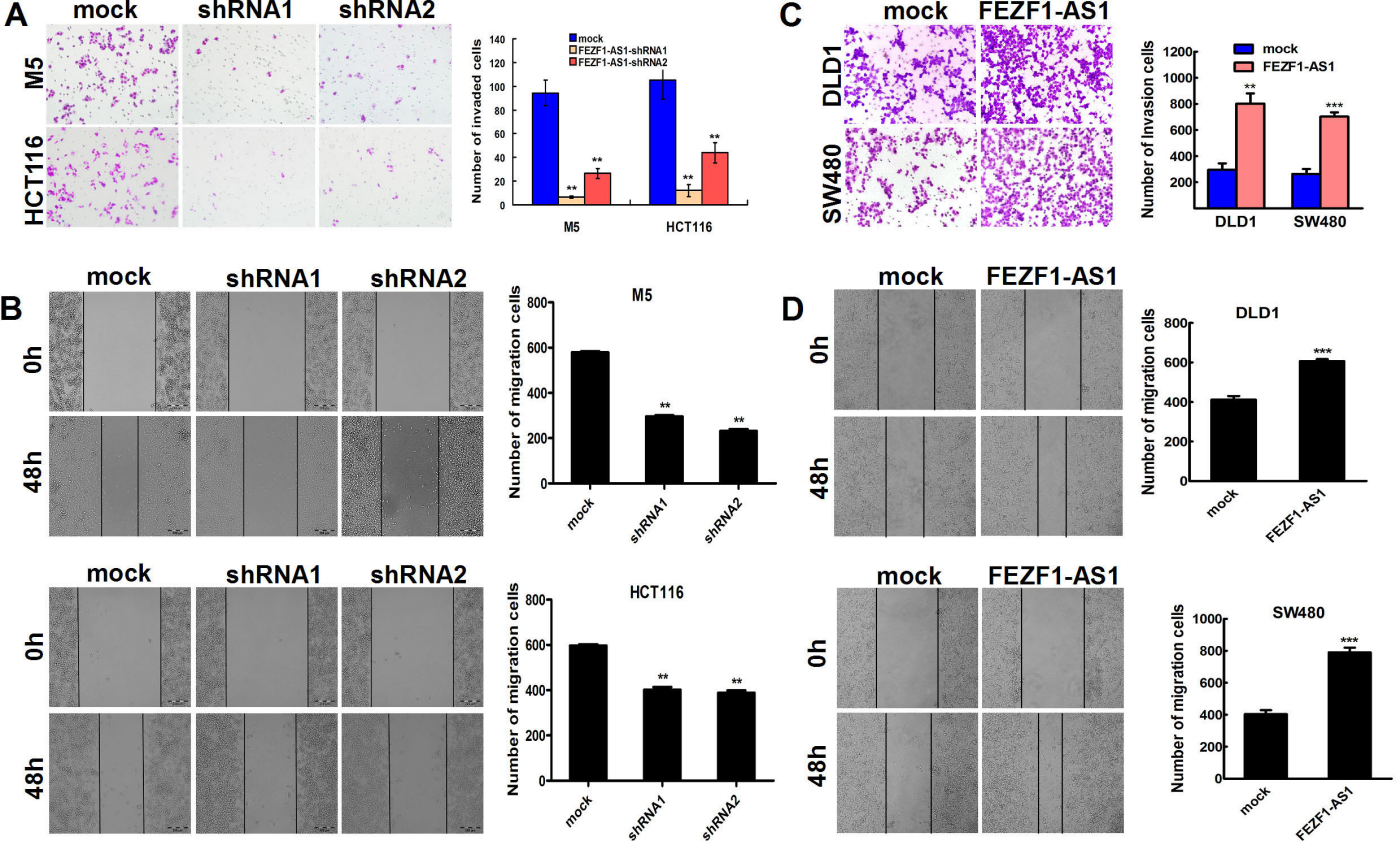
*FEZF1-AS1* promotes migration and invasion of CRC cells *in vitro* (**A**) Effects of decreased *FEZF1-AS1* expression on the migration potency of M5 and HCT116 were determined using matrigel invasion assay. (**B**) Effects of decreased *FEZF1-AS1* expression on the invasion potency of in M5 and HCT116 cells were determined using scratch-wound healing assay. (**C, D**) Effects of overexpression *FEZF1-AS1* on migration and invasion of SW480 and DLD-1 cells were determined using matrigel invasion and scratch-wound healing assay. The results were reproducible in three independent experiments. Data were presented as mean ± SD. **p < 0.001, ***p < 0.0001.

We also examined the effect of *FEZF1-AS1* knockdown on CRC cells migration/invasion. Our results showed that the *FEZF1-AS1* knockdown inhibited cell invasion by 93.0% in M5 cells, and by 88.6% in HCT116, respectively (*p* < 0.001, Figure 3A) using Matrigel invasion assay. The wound-healing assay also illustrated that *FEZF1-AS1* knockdown in CRC cells caused a significant decrease in cell migration (*p* < 0.001, Figure 3B). In contrast, invasion and migration were increased by *FEZF1-AS1* overexpression in DLD-1 and SW480 cells (*p* < 0.001, Figure 3C and 3D). These results indicated that *FEZF1*-*AS1* facilitated CRC cell migration and invasion *in vitro*.

### Knockdown of *FEZF1-AS1* inhibits tumor growth and metastasis in nude mice

**Figure 4:**
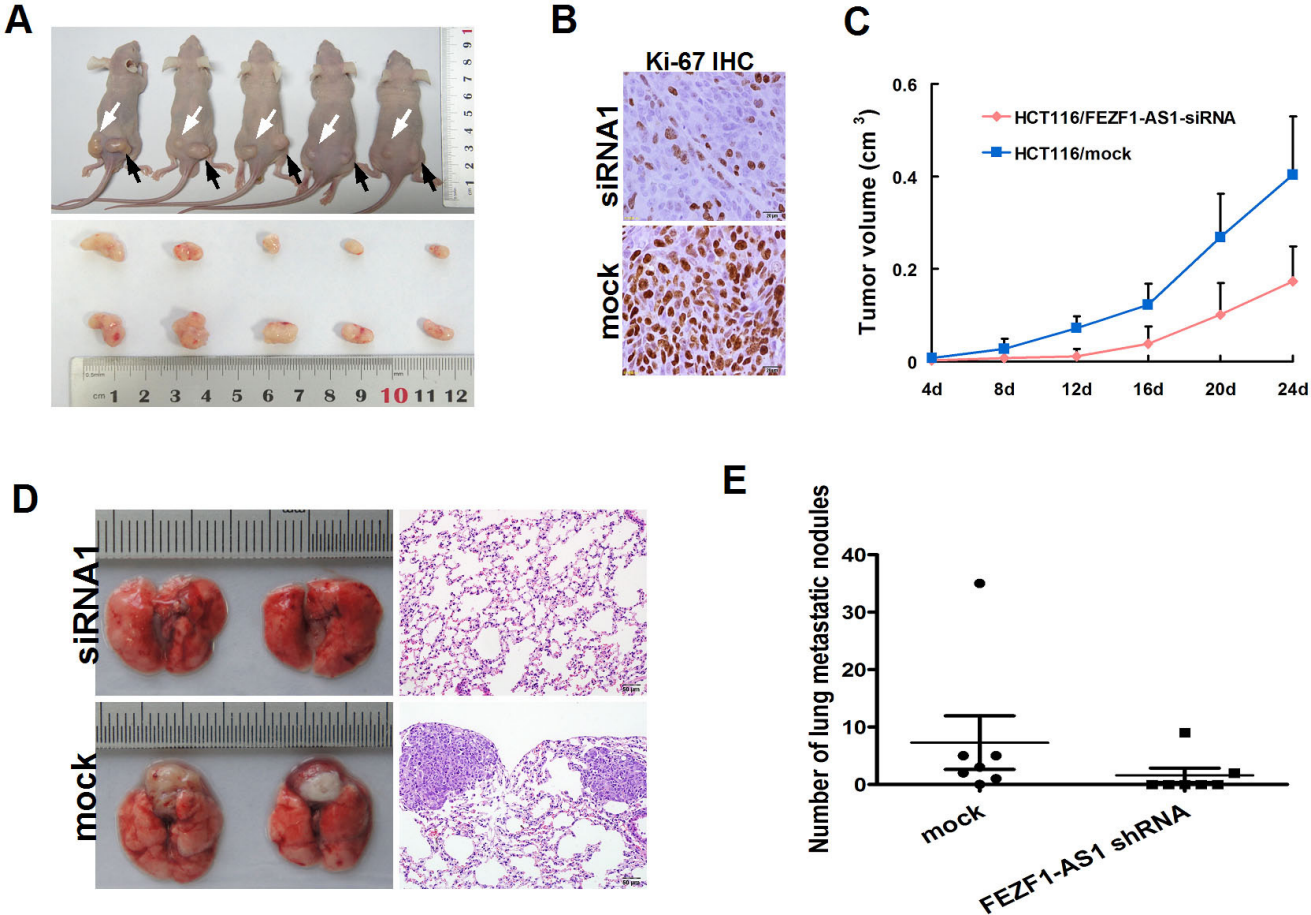
Knockdwon of *FEZF1-AS1* repressed CRC growth, invasion and metastasis *in vivo* (**A**) *FEZF1-AS1* knockdown HCT116 and mock cells injected subcutaneously into nude mice. At 24 days after subcutaneous injection, HCT116/FEZF1-AS1-siRNA1 and HCT116/mock cells produced primary tumors (upper) and representative figure of tumor formed (lower). (**B**) Immunohistochemistry showed *FEZF1-AS1* knockdown decreased the proliferation index Ki67 (×200). (**C**) Tumor growth curve of tumor volumes. Each data point represents the mean ± SD. (**D**) The whole-body images (left) and histological images (right) of metastatic nodules in lungs. (**E**) Lung metastatic nodules in individual mice were counted under the microscope.

The effect of *FEZF1-AS1* on tumor growth was assessed *in vivo* by administering a subcutaneous injection of HCT116 with stable knockdown of *FEZF1-AS1* and mock cells into the subcutaneous bilateral hind leg of mice. During a 30-day follow up period, the average tumor volume in HCT116 cells with *FEZF1-AS1* knockdown was reduced compared with mock cells. Moreover, using immunohistochemical staining of resected tumor tissues for Ki-67, we found that the proliferation index was reduced in tumor tissues formed from *FEZF1-AS1* knockdown, compared with those formed from control cells (*p* < 0.05, Figure 4A–4C).

The effect on metastasis was evaluated 8 weeks after the mice had been injected with the same cells through tail vein. We examined the number and size of tumor metastatic nodules under a microscope in the lung. As shown in Figure 4D and 4E, the number of pulmonary metastatic nodules was clearly decreased in the knockdown of *FEZF1-AS1* group compared with that of in control group (*p* = 0.036,). These results indicate that *FEZF1*-*AS1* down-regulation could inhibit tumor growth and metastasis *in vivo*.

### Konckdown of *FEZF1-AS1* decreases the expression of *FEZF1* mRNA and protein

**Figure 5:**
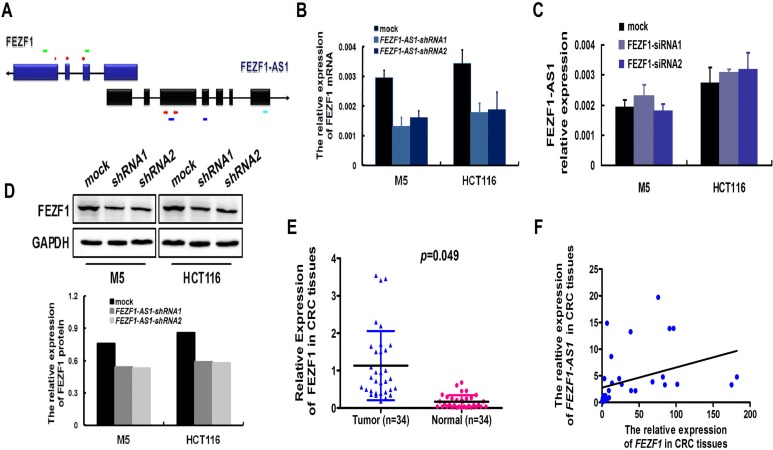
Konckdown of *FEZF1-AS1* decreased the expression of *FEZF1* (**A**) Schematic illustration of *FEZF1-AS1* and *FEZF1* structure. There are 611 nucleotides overlapping and complementary regions between the first exon of *FEZF1-AS1* and *FEZF1* exon1. Red arrowhead denoted the position of PCR primers. Light green lines denoted the position of siRNA sequence of *FEZF1* gene. Blue lines denoted the position of shRNA sequence of *FEZF1-AS1*. Light blue line denoted the location of ISH probe. (**B, D**) Levels of *FEZF1* mRNA and protein after *FEZF1-AS1*-knockdown expression in CRC cell lines examined by real-time RT-PCR (B) and western blot (D). (**C**) Levels of *FEZF1-AS1* mRNA after *FEZF1* down-regulated expression in CRC cell lines examined by real-time RT-PCR. (**E**) Expression levels of *FEZF1* in paired CRC and adjacent normal tissues by qRT-PCR. (**F**) Statistically significant positive correlation between *FEZF1-AS1* and *FEZF1* mRNA expression in CRC tissues (Spearman's correlation analysis, *r*^2^ = 0.1346).

*FEZF1-AS1* is a conserved ∼2.6-kb RNA transcribed from the plus strand of chromosome 7, on the opposite strand of the gene coding *FEZF1* protein (7q31.32), including 611 nucleotides of full complementarity between the first *FEZF1-AS1* exon and *FEZF1* exons 1 (Figure 5A). To determine the relationship between *FEZF1-AS1* and *FEZF1*, we examined the expression levels between *FEZF1-AS1* and its sense-cognate gene *FEZF1*. After knockdown endogenous *FEZF1-AS1* expression in M5 and HCT116 cells, the expression of *FEZF1* mRNA was reduced in both two cell lines. This was confirmed in protein level by western blotting (Figure 5B and 5D). However, down-regulated of *FEZF1* expression did not significantly affect *FEZF1-AS1* expression in CRC cells (Figure 5C). Furthermore, we also detected the expression levels of *FEZF1* in the same cohort of 34 paired tissue samples using real-time PCR, the results indicated that *FEZF1* expression levels in tumor tissues of CRC patients were significantly higher than those in corresponding normal tissues (*p* < 0.05, Figure 5E). At the same time, we observed a good positive correlation between *FEZF1-AS1* and *FEZF1* mRNA expression (Spearman's correlation analysis, *r*^2^ = 0.1346; *p* = 0.033, Figure 5F). These results suggested that the expression of *FEZF1* might be regulated by *FEZF1-AS1*.

### siRNA-mediated knockdown of *FEZF1* inhibited CRC cell proliferation, migration and invasion *in vitro*

**Figure 6:**
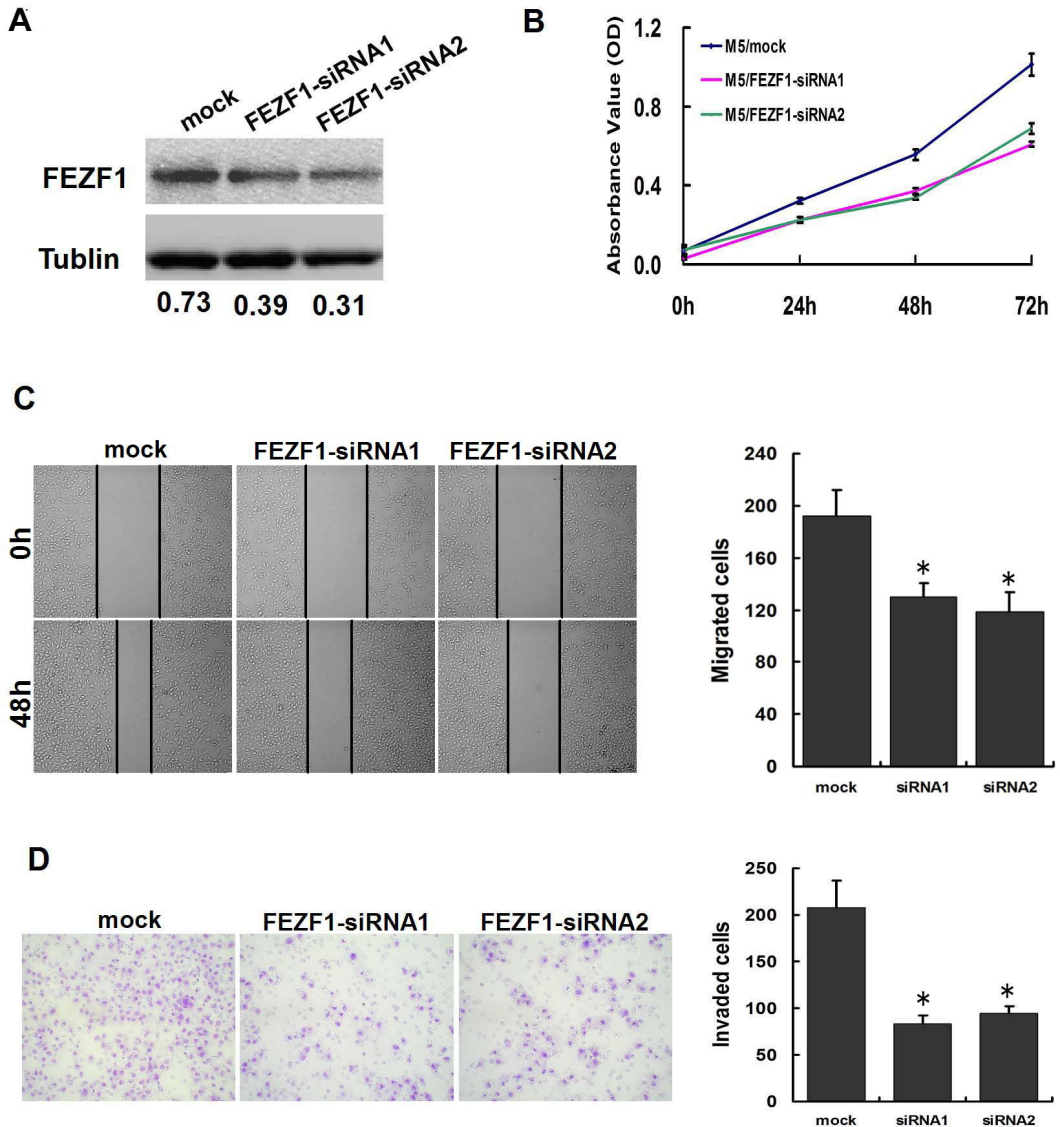
Knockdown of *FEZF1* inhibited proliferation, invasiveness and migration of CRC cells *in vitro* (**A**) Effects of *FEZF1* siRNA on *FEZF1* protein expression in M5. (**B**) The effect of *FEZF1* on cell proliferation was evaluated by CCK-8 assay after knockdown of *FEZF1* of M5. (**C**) Effects of decreased *FEZF1* expression on the migration potency of M5 were determined using scratch-wound healing assay. Data were presented as mean ± SD. The results were reproducible in three independent experiments. (**D**) Effects of *FEZF1-AS1* on invasiveness of M5 were determined using matrigel invasion assay. *p < 0.05.

We then tested whether *FEZF1* was functionally involved in CRC tumorigenesis. siRNA transfection-mediated *FEZF1* knockdown decreased cell growth relative to negative control at day 5 in both cell lines (*p* < 0.05; Figure 6A and 6B). Furthermore, we also examined the effect of *FEZF1* knockdown on M5 cells migration/invasion using the wound-healing and matrigel invasion assay. The results indicated that *FEZF1* knockdown in M5 cells caused a significant decrease in cell migration (*p* = 0.009 and 0.007, Figure 6C). Matrigel invasion assay also showed that the *FEZF1* knockdown inhibited cell invasion by 59.8% and 54.9% in M5 cells (*p* = 0.002 and 0.003, Figure 6D). These results indicated that the down-regulation of *FEZF1* expression was sufficient in reducing both cell migration and invasion *in vitro*.

## DISCUSSION

It has now become widely accepted that mammalian genomes encode numerous lncRNAs [16]. Dysregulation of some lncRNAs has been shown in various types of cancers, including CRC [5, 8, 11, 17–22]. However, the functions and the mechanisms behind lncRNAs in CRC remain obscure. We compared the different expression profiles of lncRNA between CRC and normal mucosa tissues and mined numerous lncRNAs with deregulation expression in CRC tissues (data not shown). Among those different expression lncRNAs, we chose and focused on lncRNA *FEZF1-AS1*, a previously unstudied lncRNA. To the best of our knowledge, we present the first study that evaluates the prognostic impact of *FEZF1*-*AS1* expression and comprehensive function studies of *FEZF1*-*AS1* in CRC. In this study, we found that the expression of *FEZF1-AS1* increased in CRC tissues, and CRC patients with higher *FEZF1*-*AS1* expression tend to have advanced T-stage, lymph node, or distant metastasis. Furthermore, a Kaplan-Meier analysis revealed that *FEZF1-AS1* over-expression in tumor cells had a significantly worse prognostic impact on the overall survival of CRC patients. The results indicated that *FEZF1-AS1* could serve as a predictor for advanced CRC and poor prognosis for CRC patients.

In order to highlight the function of up-regulation of *FEZF1-AS1* in CRC, we further explored the critical role of *FEZF1-AS1* in the progression of CRC by loss-of-function and gain-of-function experiments. Our results revealed that the reducing of *FEZF1*-*AS1* expression could significantly inhibit CRC cells proliferation, migration and invasiveness, and suppress S-phase entry *in vitro*. In contrast, overexpression of *FEZF1-AS1* could promote the aggressive behaviors of CRC cells. Furthermore, the downregulation of *FEZF1-AS1* could inhibit the tumorigenesis and lung metastasis in murine model. Taken together, these findings suggested that *FEZF1-AS1* might serve as an oncogene and play a promotion role in CRC development and progression. Many other lncRNAs have also been implicated in the development of multiple tumors and identified as cancer biomarkers. Examples include promotion of oesophageal adenocarcinoma cells invasion and metastasis by *HNF1A-AS1*, control of colorectal cancer cells apoptosis by *ZFAS1*, reprogramming of induced pluripotent stem cells by *RNA-RoR*, and regulation of non-small cell lung cancer cells growth and apoptosis by *MEG3* [22–25]. Thus, effective blocking of these lncRNAs in cancer could be a novel preventive and therapeutic strategy.

The importance of lncRNAs in human cancer may be associated with their abilities to impact cellular functions through various mechanisms. Previous studies have demonstrated that the mechanisms of lncRNAs are partly dependant on their genomic location [26, 27]. It is noteworthy that some lncRNAs, which are oriented in antisense direction with respect to a protein coding loci in the opposite strand, usually act as regulator of this gene [28, 29]. *FEZF1-AS1* is localized in the antisense DNA strand of the *FEZF1* gene, which has been identified as critical roles in gastric cancer cell proliferation and tumorigenesis by transcriptional activation of the *K-ras* gene [30, 31]. Therefore, we hypothesized that *FEZF1-AS1* might regulate *FEZF1* to contribute to progression of malignant diseases. Further experiments revealed the knockdown of *FEZF1-AS1* reduced *FEZF1* mRNA and protein expression in CRC cells, and that the expression levels of *FEZF1* were significantly higher in tumor tissues. Consistent to these finding, there was a positive correlation between *FEZF1-AS1* and *FEZF1* sense expression in CRC. These data showed that *FEZF1* mRNA expression is under the control of lncRNA *FEZF1-AS1.* Furthermore, we validated that *FEZF1* knockdown also significantly suppressed CRC cell proliferation, invasion, and migration, but did not affect the expression of *FEZF1*-*AS1*. Our observations indicated that the promotion effects of *FEZF1*-*AS1* on CRC might be achieved partly by activating *FEZF1* expression. Because of including 611 nucleotides of full complementarity between the exon of *FEZF1-AS1* and *FEZF1*, we used an RNase protection assay to test the possibility of RNA duplex formation to analysis the mechanism that *FEZF1-AS1* regulated *FEZF1* expression. However, the results did not validated that *FEZF1-AS1* formed RNA duplex with *FEZF1* mRNA and increased stability of *FEZF1* mRNA (data not shown). So, further studies are needed to elucidate the precise mechanisms by which *FEZF1*-*AS1* modulates its targets.

In summary, this study provides the first link between *FEZF1-AS1* expression and CRC development. We proved that the *FEZF1-AS1* was upregulated in CRC tissues and its increased expression might play a promotion role in CRC tumorigenesis and progression. These results indicate that *FEZF1-AS1* might be a candidate prognostic biomarker and a target for new therapies of CRC.

## MATERIALS AND METHODS

### Cell culture

The human embryonic kidney cells 293T and the human CRC cell lines DLD-1, HT29, HCT116, SW480, SW620, Lovo were obtained from a cell bank at the Chinese Academy of Sciences (Shanghai, China). In previous studies, we described a subclone named M5 with enhanced metastatic abilities in liver [32, 33]. All CRC cell lines were cultured in RPMI 1640 medium (Gibco, Gaithersburg, MD, USA) with 10% fetal bovine serum (HyClone, Logan, USA) and 100 U/ml penicillin/streptomycin (Gibco). They were maintained in a humidified chamber containing 5% CO_2_ at 37°C. 293T was maintained in Dulbecco's modified Eagle's medium (DMEM) that was supplemented with 10% FBS.

### Tissue preparation

Fresh and formalin-fixed, paraffin-embedded, colorectal tumor tissue samples were obtained from patients who were diagnosed with primary CRC. Elective surgery was carried out on these patients at Nanfang Hospital, Southern Medical University (Guangzhou, China). In total, 34 cases of fresh CRC tissue were freshly frozen in liquid nitrogen and stored at −80°C until further use. 153 cases of archived, formalin-fixed, paraffin-embedded CRC tissue samples were collected and used in clinicopathological and prognostic investigation of *FEZF1-AS1*. A comprehensive set of clinicopathological data were recorded, including age, gender, size of primary tumor, tumor differentiation, T stage, lymph node metastasis, and distant metastasis. The stage of disease was determined according to the tumor size, lymph node, and metastasis (pTNM) classification system [34]. Complete follow-up, ranging from 1–96 months, was available for the cohort of 153 patients, and the median survival was 56 months. The use of tissues for this study has been approved by the ethics committee of Nanfang Hospital, Southern Medical University. Before using these clinical materials for research purposes, all the patients signed the informed consent. None of these patients received any pre-operative chemotherapy or radiotherapy.

### RNA isolation and quantitative real-time PCR

Total RNA was extracted using TRIzol Reagent (Invitrogen, Carlsbad, CA). cDNA was synthesized using the PrimeScript RT reagent Kit (Promega, Madison, WI, USA). Quantitative real-time RT-PCR was performed with the SYBR Premix EX Taq^™^ (Takala, Dalian, China) using an ABI 7500 Real-Time PCR system (Applied Biosystems, Foster City, USA). *GAPDH* was used as an endogenous control. Fold changes were calculated through relative quantification (2^−ΔΔCt^). Primers of *FEZF1-AS1*: 5′-TTAGGAGGCTTGTTCTGTGT-3′ and 5′-GCGCAGGTACTTAAGAAAGA-3′ (aligns to nucleotides 901–920 and 1120–1139 of the *FEZF1-AS1* Reference Sequence NR_036484, respectively). *GAPDH*: 5′-ACAGTCAGCCGCATCTTCT-3′ and 5′-GACAAGC-TTCCCGTTCTCAG-3′ (aligns to nucleotides 134–152 and 465–485 of the *GAPDH* Reference Sequence NM_001289745, respectively). *FEZF1*: 5′-CAGGCACAA GATCATTCACACGCAG-G-3′ and 5′-CCCTTTTTGA TGAAACCCTTTGCCACAG-3′ (aligns to nucleotides 986–1011 and 1118–1145 of the *FEZF1* Reference Sequence NM_001024613, respectively). To account for the assessment of technical variability, the assay was performed in triplicate for each case.

### *In situ* hybridization (ISH) and evaluation of staining of *FEZF1-AS1*

*FEZF1-AS1* expression was examined using *in situ* hybridization (ISH) in CRC and non-CRC paraffin-embedded sections. Briefly, after dewaxing and rehydration, the samples were digested with proteinase K, fixed in 4% paraformaldehyde, hybridized with the 5′digoxin-labeled probe 5′-TTCGACTGTTTCCTTGACACTAC-3′ (aligns to nucleotides 2550–2572 of the *FEZF1-AS1* Reference Sequence NR_036484) at 55°C overnight, and subsequently incubated for 30 minutes at 4°C with HRP. Diaminobenzidine (DAB) was used for performing color reactions. An unrelated sequence that does not match any known human gene was used as negative control.

The positive expression of *FEZF1-AS1* was primarily detected in the cytoplasm. To assess the clinical characteristics of patients, the ISH stained tissue sections were reviewed and scored separately by two blinded pathologists. Based on both the intensity and proportion of *FEZF1-AS1*-positive cells, the staining scores were determined using a relatively simple, reproducible scoring method [33, 35]. On a scale of 0 to 3, the staining intensity was scored as follows: 0 (no staining), 1 (weak), 2 (medium), or 3 (strong). The extent of staining is defined as the percentage of positive staining areas of tumor cells or normal colonic epithelial cells in relation to the whole tumor area or entire section for normal samples. The extent of staining was scored on a scale of 0 to 4 as follows: 0, 0%; 1, 1–25%; 2, 26–50%; 3, 51–75%; and 4, 76–100%. The sum of the staining-intensity and staining-extent scores was used as the final staining score for *FEZF1-AS1* (0–7). For statistical analysis, a final staining score of ≥ 3 was considered to be high.

### Vector preparation

To reduce *FEZF1-AS1* expression, two human *FEZF1-AS1* shRNA sequence 5′-GCACG CTTCCGAGTTTCCATT-3′ and 5′-GCCTGATGTCTA ACAGAAAGG-3′ (aligns to nucleotides 1079–1099 and 1869–1889 of the *FEZF1-AS1* Reference Sequence NR_036484) was respectively cloned into pGU6/GFP/Neo-shRNA (GenePharme, Shanghai, China). Thereafter, two *FEZF1-AS1* knockdown vectors, named pGU6/GFP/Neo-*FEZF1-AS1*-shRNA1 and pGU6/GFP/Neo-*FEZF1-AS1*-shRNA2 were constructed. A scrambled shRNA oligo namely pGU6/GFP/NC-Neo-shRNA, which does not match any known human gene, was used as a control. The sequence of *FEZF1-AS1* was synthesized and subcloned into pcDNA3.1 (Invitrogen, Shanghai, China). Ectopic expression of *FEZF1-AS1* was achieved by using the pcDNA-*FEZF1-AS1* transfection and empty pcDNA vector (empty) was used as control. The expression level of *FEZF1-AS1* was detected by qPCR.

Two human small interfering RNAs (siRNAs) targeting *FEZF1* (N1, 5′-GGGTTTCTGCAGGAACT TTGA-3′ and N2, 5′-GCACAAGATCATTCACACGCA-3′, aligns to nucleotides 989–1009 and 1295–1315 of the *FEZF1-AS1* reference sequence NR_036484, respectively) and a human scrambled siRNA sequence possessing limited homology with human genes was used as a negative control were synthesized.

### Lentivirus production and construction of stable cell lines

Virus particles were harvested 48 hours after pGU6/GFP/Neo-*FEZF1-AS1*-shRNA1or-shRNA2 transfection with the packaging vector pG-P1-VSVG, pG-P2-REV and pG-P3-RRE into 293T cells using lipofectamine 2000 reagent (Invitrogen). HCT116 and M5 cells were infected using the recombinant lentivirus-transducing units plus 8 mg/ml Polybrene (Sigma, St Louis, Missouri, USA). Then, they were subjected to FACS analysis for GFP expression to gain CRC cells with stable knockdown of *FEZF1-AS1*.

### Transfection of colon cancer cells

DLD-1, SW480, HCT116 and M5 cells were seeded into six-well plates. DLD-1 and SW480 cells were transfected with the pcDNA-*FEZF-AS1* or empty vector, two *FEZF1* siRNAs and control siRNA then were transfected into the HCT116 and M5 cells using Lipofectamine (Invitrogen, Carlsbad, CA) according to the manufacturer's instructions, Cells were harvested after 48 hours for qRT-PCR and western blot analyses.

### Cell proliferation assay

2-(2-Methoxy-4-nitrophenyl)-3-(4-nitrophenyl)-5-(2,4-disulfothenyl)-2*H*-tetrazolium salt (CCK-8, Dojindo, Rockville, USA) assay was conducted to evaluate the rate of cell proliferation, according to manufacturer's instructions. Briefly, log-phase cells were trypsinized into a single-cell suspension and plated into 96-well plates at a density of 2 × 10^3^ per well. CCK-8 solution was added to each well. After 1 hour, the absorbance of each well was recorded at 450 nm and read on a microplate reader victor (Enspire 2300 Maltilabel Reader, PerkinElmer, Singapore)

### Colony formation assay

The cells were plated in 6-well plates at 2 × 10^2^ per well and maintained in RPMI1640 containing 10% FBS for 2 weeks. After 2 weeks, the cells were washed twice with PBS, fixed with methanol and stained with 0.5% crystal violet. The number of colonies whose diameter was greater than 150 μm was counted under a microscope [36].

### Cell migration assay

Cell motility was measured with wound healing assay by measuring the movement of cells in a scraped, acellular area created by a 200 μL pipette tube, and the spread of wound closure was observed after 0 and 48 hours, respectively. Photographs were taken to assess the level of migration in each group of transfected cells. The migration was quantified by counting the total number of cells that migrated toward the original wound field.

### Cell invasion assay

For invasion assay, matrigel-coated chambers (BD Biosciences, San José, CA, USA) containing 8 μm pores were used. Cells were seeded in the upper chambers (coated in matrigel) at 2 × 10^5^ concentration in serum-free medium. The lower chamber of the transwell was filled with culture media containing 10% FBS as a chemo-attractant. After these chambers were incubated at 37°C for 48 hours, non-invaded cells on the top of the transwell were scraped off with a cotton swab. The successfully translocated cells were fixed with 10% formalin. Then, they were stained with 0.1% crystal violet for 30 minutes and counted under a light microscope.

### Flow cytometric analysis

Cells were seeded at a density of 1 × 10^6^ cells/well in six well plates. After 24 h, cells were washed with PBS and fixed in ice-cold 70% ethanol for 1 h and then treated with 100 μL of 50 mg/L propidium iodide for 30 min at 4°C in the dark. The cell cycle profiles were assayed using the Elite ESP flow cytometer at 488 nm, and data were analyzed with the CELL Quest software (BD Biosciences, San Jose, CA, USA).

### Animal experiments

Balb/C-nu/nu athymic nude mice (4–6 weeks old) were obtained from the Laboratory Animal Centre of Southern Medical University. For tumor growth assay, a total of 2 × 10^6^ cells suspended of HCT116 with stable knockdown of *FEZF1-AS1*, or mock cells, were respectively injected into left and right bilateral hind leg subcutaneous of mice. The tumor size was measured using digital calipers every three days. After 24 days of monitoring, mice were sacrificed by cervical dislocation and tumors were dissected. Tumor volume was calculated as follows: Volume = (D × d^2)^/2, where D meant the longest diameter and d meant the shortest diameter.

For the *in vivo* metastasis assay, the mice were *i.v.* injected via the lateral tail vein with 2 × 10^6^ tumor cells or control cells were suspended in 0.1 mL of culture medium. Animal health status was monitored every three days. Human endpoints had been planned at the end of experiment as a means to relieve pain or distress. At the time of euthanasia, the lungs were removed by dissection away from adjacent organs and fixed with 10% neutral buffered formalin. Subsequently, the consecutive tissue sections were obtained and stained with haematoxylin-eosin (H & E) to observe the metastatic nodules of lungs under microscope. All the experimental procedures were performed in strict accordance with the recommendations in the Guide for the Care and Use of Laboratory Animals of the National Institutes of Health. The protocol was approved by the Committee on the Ethics of Animal Experiments of Southern Medical University. All the necessary steps were taken to minimize the suffering and distress caused to the mice.

### Statistical analysis

All statistical analyses were performed using the SPSS 16.0 statistical software package. In at least three independent experiments, the data were presented in terms of mean ± SEM. The differences between variables were assessed using three statistical tests: χ^2^ test, Fisher's exact test, or One-way ANOVA. For patients with different levels of *FEZF1-AS1* expression, the survival curves were plotted using the Kaplan–Meier method and compared using the log-rank test. The multivariate survival analysis was performed on all parameters that were found to be significant in univariate analysis using the Cox regression model. A *p* value less than 0.05 was considered as statistical significance.
